# Genomic and spatial analysis of local recurrences following risk-adapted breast radiotherapy in the IMPORT trials

**DOI:** 10.1186/s13058-026-02232-9

**Published:** 2026-04-25

**Authors:** Sara V. Lightowlers, Maria Roman-Escorza, Ceilidh Welsh, Judith M. Bliss, Jason S. Carroll, H. Y. Charlie Chan, Karl Harrison, Joanne S. Haviland, Nuala Healy, Monica L. Jefford, Rajesh Jena, Anna M. Kirby, Cliona C. Kirwan, Elena Provenzano, Mark A. Sydenham, Isabel Syndikus, James Tanner, Ramona Woitek, Charlotte E. Coles, Elinor J. Sawyer

**Affiliations:** 1 Department of Oncology, University of Cambridge, Cambridge, UK; 2 School of Cancer and Pharmaceutical Sciences, Faculty of Life Sciences and Medicine, Guy’s Cancer Centre, King’s College London, London, SE1 9RT UK; 3 The Institute of Cancer Research Clinical Trials and Statistics Unit, Institute of Cancer Research, London, UK; 4 Cancer Research UK Cambridge Institute, Robinson Way, Cambridge, CB2 0RE UK; 5 Department of Breast Surgery, Nuffield Health Cheltenham Hospital, Cheltenham, UK; 6 Cavendish Laboratory, University of Cambridge, Cambridge, CB3 0HE UK; 7 Wolfson Institute of Population Health, Queen Mary’s University, London, UK; 8 Cambridge Breast Unit, Cambridge University Hospital NHS Foundation Trust, Cambridge, UK; 9 Department of Radiology, Beaumont Hospital, Dublin, Ireland; 10 Department of Radiology, Royal College of Surgeons in Ireland, Dublin, Ireland; 11 Independent Cancer Patients Voice, London, UK; 12 Breast Unit, Royal Marsden NHS Foundation Trust and Institute of Cancer Research, Sutton, UK; 13 Division of Cancer Sciences, University of Manchester, Manchester, UK; 14 NIHR Cambridge Biomedical Research Centre, Cambridge University Hospitals NHS Foundation Trust, Cambridge, UK; 15 Department of Histopathology, Addenbrookes Hospital, Cambridge, UK; 16 Department of Radiotherapy, Clatterbridge Cancer Centre, Bebington, UK; 17 Department of Radiology and Cancer Research UK Cambridge Center, Cambridge, CB2 0QQ UK; 18 Research Center for Medical Image Analysis and Artificial Intelligence (MIAAI), Danube Private University, 3500 Krems, Austria; 19 Guys Cancer Centre School of Cancer and Pharmaceutical Sciences, Guy’s and St Thomas’ NHS Foundation Trust, Kings College, London, UK

**Keywords:** Breast cancer, Breast radiotherapy, Risk-adapted radiotherapy, Local recurrence, Tumour evolution

## Abstract

**Background:**

Ipsilateral breast tumour recurrences (IBTR) that occur after breast-conserving surgery (BCS) and breast radiotherapy may be ‘true recurrences’ or independent ‘new primaries’. Contralateral breast cancers (CBC) are usually assumed to be new primary tumours. Understanding the patterns of occurrence of these entities, and/or being able to reliably distinguish between the two, could have important implications for tailoring patient management at primary presentation and at IBTR/CBC diagnosis.

**Methods:**

The clonal relationship between index, and ipsilateral and contralateral subsequent breast tumours was assessed in 112 paired samples from two breast radiotherapy trials, IMPORT LOW and IMPORT HIGH, using copy number profiling and targeted sequencing of DNA. The spatial relationships between the index tumour beds, radiotherapy dose distributions and subsequent tumours were analysed via computational co-registration of cross-sectional imaging.

**Results:**

In the IMPORT HIGH cohort, where patients were at higher risk of IBTR, 61% of IBTRs were clonally related to the index tumour compared with only 32% from the IMPORT LOW cohort. There was no difference in the spatial distributions relative to index tumour beds of related compared to unrelated subsequent tumours. Related IBTRs occurred in a higher proportion of those diagnosed less than 5 years after index treatment, compared with those diagnosed after 5 years (66% vs. 24%). Four of 18 CBCs in the IMPORT HIGH cohort, but none from IMPORT LOW, were genomically similar to the index cancers.

**Conclusions:**

These findings show that IBTRs near the index tumour bed are not always true recurrences and also strongly suggest that some CBCs are metastases.

**Supplementary Information:**

The online version contains supplementary material available at 10.1186/s13058-026-02232-9.

## Introduction

Following breast-conserving surgery (BCS) and whole breast radiotherapy, reported rates of ipsilateral breast tumour recurrence (IBTR) vary from 5 to 15% over 10 years [1–3]. Previous reports have suggested that the majority of IBTRs seem to occur near the original tumour bed [4] and that these may represent ‘true recurrence’ (derived from residual malignant cells of the index cancer that have not been sterilized by radiotherapy). A smaller number occur elsewhere in the breast and are thought to be ‘new primaries’ (independently occurring cancer). Factors such as the timeframe between the index and subsequent tumours and pathological factors (grade, receptor status) may also influence whether IBTR is considered a true recurrence or not. However, there are few molecular data to support these assumptions.

The distinction between the two entities is important as true recurrences could be hypothesised to have a worse prognosis than new primaries, as they have resisted previous therapies, and may therefore require more intensive treatment. However, there is no gold standard method of distinguishing between the two, and consequently, this hypothesis has been difficult to test. Several reports have used varying clinical classification systems to differentiate true recurrence from new primary, based on factors such as location and concordance of grade and receptor status. An analysis comparing six of these classification systems [5] found that each designated different proportions of the cohort of 234 cases as true recurrence/new primary and none found statistically significant differences in outcome between the two groups. Other studies have used molecular techniques to classify IBTR [6, 7]. More recently next-generation sequencing technologies have examined clonal relationships in similar contexts including multifocal [8] and synchronous bilateral breast cancers [9, 10] by comparing somatic mutations or copy number alterations.

The IMPORT LOW [11] and IMPORT HIGH [12] trials investigated the adaptation of radiotherapy dose-volume to the spatially varying risk of recurrence, testing partial breast irradiation and simultaneous integrated boost in patients with tumours at low and high risk of IBTR, respectively. These trials have been practice changing internationally [13–16]. The two large cohorts of patients with mature long-term follow-up, together with comprehensive imaging and biosample datasets, provide a unique opportunity to comprehensively interrogate the biology of IBTR. This analysis examined the genomic relationships between paired index and subsequent tumours reported as IBTR in the trials (alongside contralateral subsequent breast cancers expected to be unrelated), to define the subsequent tumours as true recurrences or unrelated new primaries. Computational co-registration of cross-sectional imaging of the index tumour bed and ipsilateral recurrence was then used to analyse the spatial relationships between the index tumours and the IBTRs.

## Methods

### Case identification

The IMPORT LOW trial recruited women following BCS, with pT1-2 pN0-1 ductal carcinomas who were considered suitable for partial breast radiotherapy and IMPORT HIGH recruited women following BCS with pT1-3 pN0-3a carcinomas who required tumour bed boost radiotherapy. Full details of eligibility criteria are available in Appendix 1, and full details of both trials have been published [11, 12]. At the time of enrolment, between 2007–2010 and 2009–2015 respectively, participants had the option to consent to the use of their tissue for future research. Those who had consented and were subsequently reported to have an IBTR or a contralateral new primary breast cancer were identified via review of case report forms (CRFs) within the clinical database.

### Clinicopathological data

Clinicopathological data were obtained from the trial database and central pathological review conducted by the trial pathologist (EP) (see supplementary data for more details).

To categorise ipsilateral subsequent tumours as being related or unrelated to the index using histological criteria we used the ‘morphology method’ described by Jobsen et al. [5]. This method was chosen as it was one of the best discriminators of outcome in that analysis and also used only histological rather than spatial criteria. Specifically, in this method histological type, ER status (positive/negative), HER2 status (positive/negative) and tumour grade had to be consistent between original and subsequent tumours for the latter to be considered a true recurrence (only a change in grade from 1 to 3 or vice versa was considered inconsistent).

Outcome data were obtained from the trial database on 28th September 2023. This included time between randomisation and diagnosis of IBTR/contralateral new primary, diagnosis of metastatic breast cancer, death, cause of death, and time from randomisation to metastatic breast cancer diagnosis or death.

### DNA sequencing

DNA was extracted as per standard methods (see supplementary data). Genomic libraries were prepared using the Nonacus Cell3 Target kit. DNA underwent shallow whole genome sequencing (sWGS) and targeted deep sequencing of a panel of cancer-related genes. Sequencing was on either an S4 flow cell on the Illumina NovaSeq 6000 system or a P2 or P3 flow cell on the Illumina NextSeq 2000 system, in all cases using 100 base pair paired-end mode. For the IMPORT LOW cohort, the targeted deep sequencing used a custom panel of cancer-related genes (see Appendix). For the IMPORT HIGH cohort, the PanCancer panel from Informed Genomics was used (see Appendix). For both cohorts, the intended sequencing depth was 0.1 × for shallow whole genome sequencing (sWGS) and 500 × for deep sequencing. The bioinformatic analysis is described in the supplementary data.

### Breakclone analysis

Relatedness of tumour pairs was determined using the R package Breakclone [17]. Breakclone calculates concordance scores separately for copy number and mutation data. For copy number data, the approach uses individual copy number aberration breakpoint position, and for mutation data, the approach uses both allele frequency and population frequency of variants in calculating the concordance score. P values for concordance scores are then calculated using a reference distribution of artificial pair concordance scores, using all possible unrelated pairs of tumours from separate patients. The p value cut off for relatedness was 0.01; pairs were considered unrelated if p ≥ 0.05, and ambiguous if between 0.01 and 0.05. In most cases, Breakclone copy number and mutation analyses gave the same verdicts. In those that were not concordant if the verdicts were ‘related’ and ‘ambiguous’, the pair were called related; if ‘unrelated’ and ‘ambiguous’, the pair were called unrelated; if ‘related’ and ‘non-related’, the pair were called ambiguous.

### Spatial data

Radiotherapy planning CT datasets from patients in IMPORT LOW and IMPORT HIGH who had experienced IBTR were obtained from the UK Radiotherapy Trials Quality Assurance Group (RTTQA). For cases in which a staging CT had been performed at the time of IBTR (recurrence CT), these were also requested from trial centres. IBTR volumes were outlined semiautomatically (i.e. using a combination of human and computational steps) on these images by a subspecialty trained consultant breast radiologist (RW, JT, or NH) using the Microsoft Radiomics App v1.0.28434.1 (project InnerEye; Microsoft, Redmond, WA, USA; https://www.microsoft.com/en-us/research/project/medical-image-analysis). To classify patterns of locoregional failure, a deformable image registration (DIR) workflow was performed using Elastix software [18] as part of the scikit-rt Python package [19] (see supplementary data).

The site of the index tumour and IBTR data was also collected on the clinical CRFs and used to determine the ‘Case Report Form spatial classification’ of either the index quadrant, if both CRF reported the same quadrant, or the distant breast if the two reports were discordant.

Researchers were blinded to clinical and spatial information prior to results of the genomic analysis.

### Statistical analyses

There was no sample size calculation; all cases that fulfilled the inclusion criteria were used in the analysis. Given the lower-than-expected event rate in both trial cohorts, the number of potential cases for this analysis was consequently low. Fisher’s Exact test was performed to assess the association between the trial cohort and the likelihood of an ipsilateral subsequent tumour being clonally related. Mann Whitney U test was performed to assess the association between ipsilateral subsequent tumour relatedness and time from trial randomisation to diagnosis of subsequent tumour. Statistical methods used in additional genomic analyses are described in the supplementary data.

## Results

**Fig. 1:**
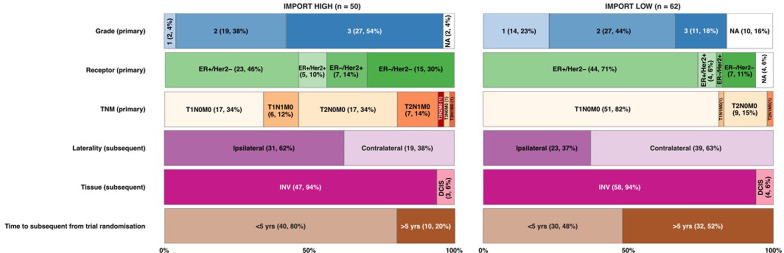
Details of cases from both trial cohorts included in this analysis

Tumour DNA was available for 112 pairs of index and subsequent tumours of which 53 were ipsilateral events (including 4 cases in which there were 2 subsequent tumours) and 59 were contralateral events. 62 of the pairs were from IMPORT LOW and 50 from IMPORT HIGH (Fig. 1). Corresponding germline DNA extracted from paired blood samples was available for 94/112 (84%). 107 tumour pairs (from 105 patients) passed QC and were included in the genomic analysis. Due to the different eligibility criteria for each trial the index tumours from the IMPORT HIGH cohort were more commonly grade 3, ER negative, HER2 positive and node positive, compared with index tumours of the IMPORT LOW cohort.

### Clonal relatedness analysis

Clonal relatedness was assessed using Breakclone, a statistical approach (Methods) that takes population frequency of different aberrations into account giving greater weight to true clonal events while down-weighting aberrations characteristic of certain cancer types that are more likely to recur independently across different tumours.

**Fig. 2:**
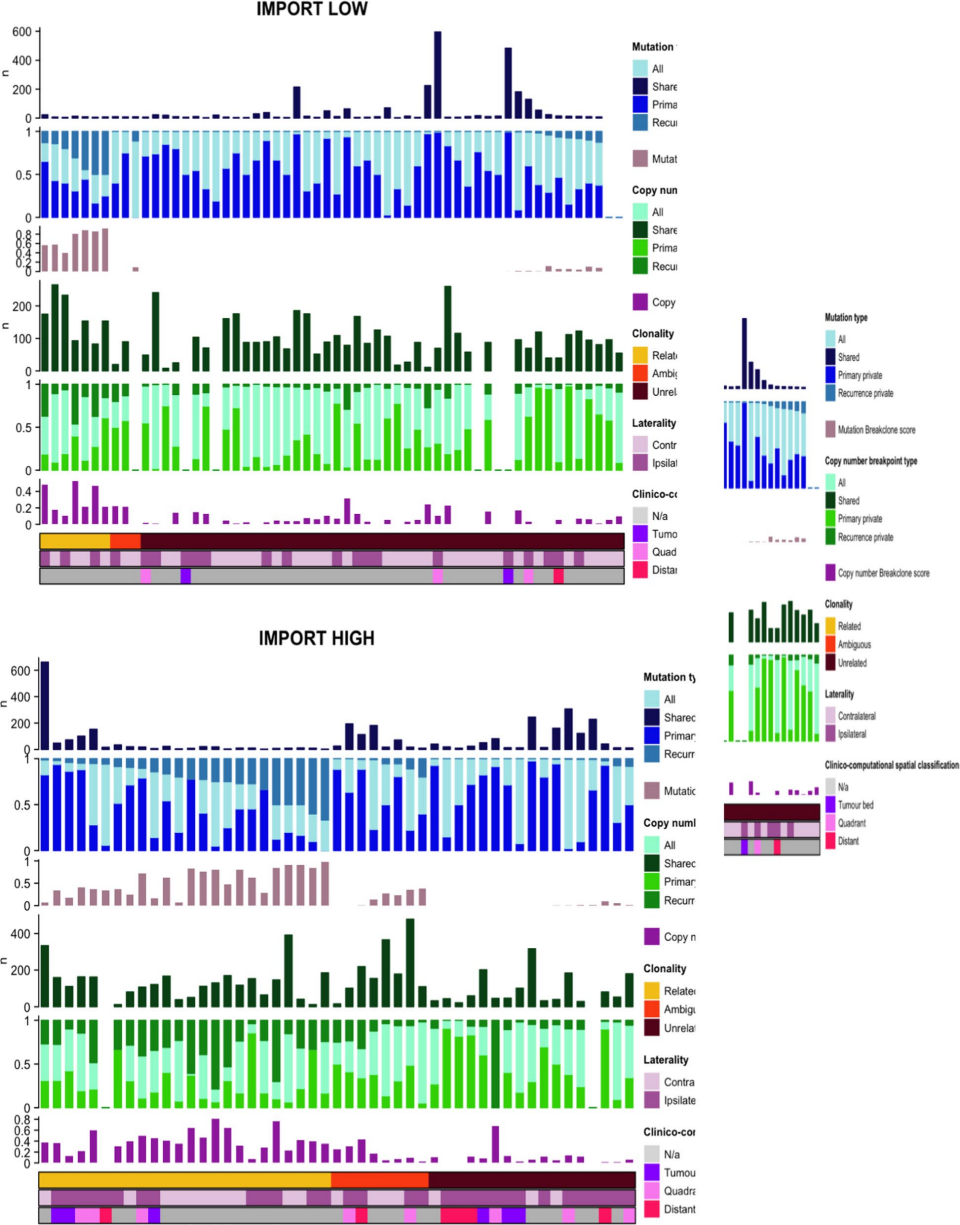
Summary of genomic data, laterality, and spatial classification of ipsilateral recurrences using computational and clinical data, in both IMPORT trial cohorts. The top barplot in each figure (“Total mutations”) indicates the combined total number of mutations in both tumours of the pair; the second barplot (“Proportion of mutations”) indicates the proportion of this total that are shared between the two tumours, private to the index tumour or private to the subsequent tumour; and the third barplot (“Mutation Breakclone score”) indicates the mutation Breakclone score for the pair. The fourth barplot (“Total breakpoints”) indicates the combined total number of breakpoints in both tumours of the pair; the fifth barplot (“Proportion of breakpoints”) indicates the proportion of this total that are shared between the two tumours, private to the index or private to the subsequent tumour; and the bottom barplot (“Copy number Breakclone score”) indicates the copy number Breakclone score for the pair. The three bars at the bottom indicate, respectively, the overall relatedness verdict using both mutation and copy number Breakclone scores, the laterality of the subsequent tumour with respect to the index tumour, and the clinico-computational spatial classification of the subsequent tumour using the deformable image registration workflow followed by clinical review

**Fig. 3:**
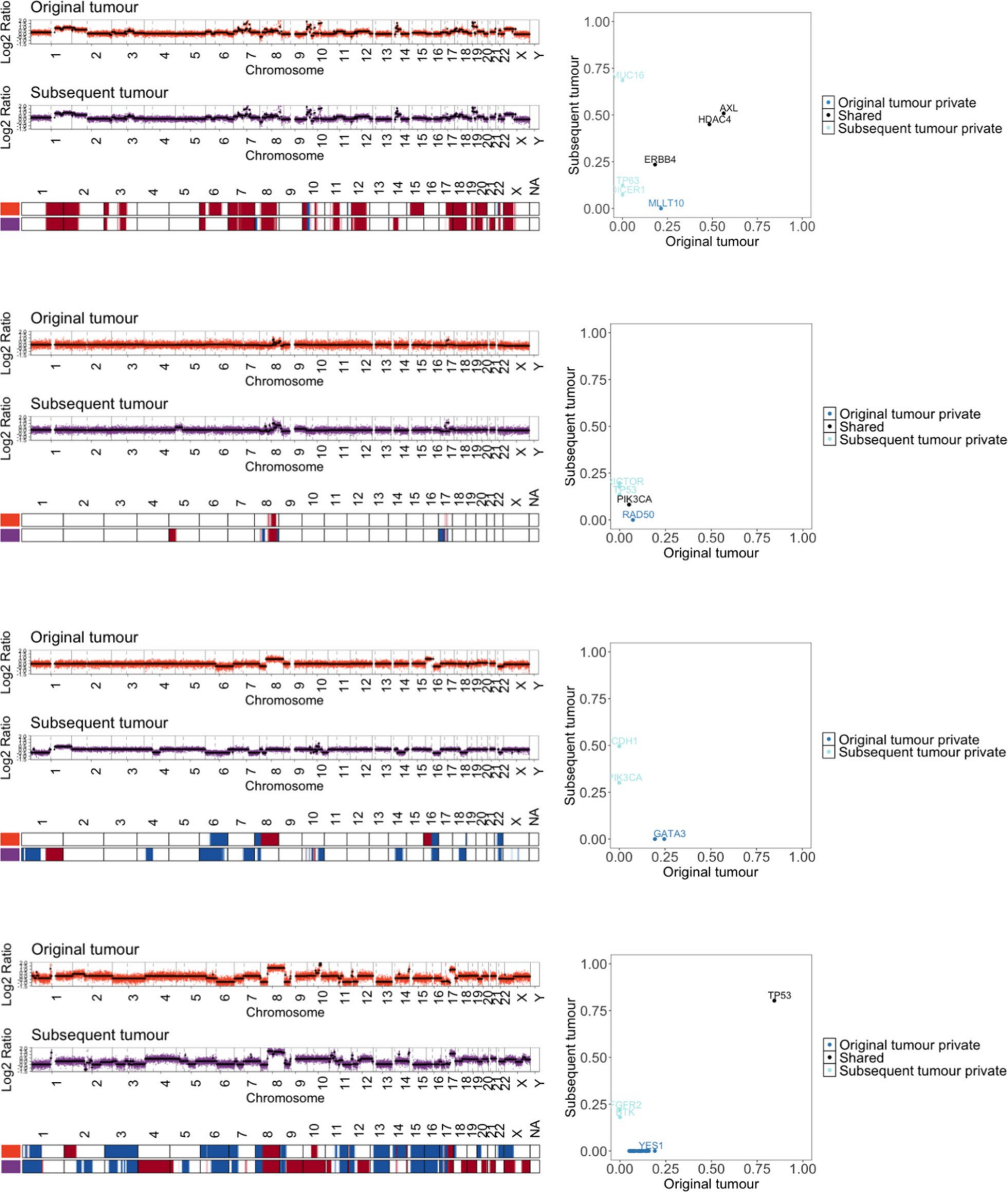
**A** Matched original and subsequent tumour copy number profiles, and **B** Plots of variant allele fraction of shared and private mutations between the paired tumours in cases genomically classified as (1) related (ipsilateral); (2) ambiguous (ipsilateral); (3) unrelated (ipsilateral); and (4) related (contralateral)

Data was available for 107 tumour pairs from 105 patients; in 5 pairs from 4 patients sequencing results were not analysable. In 73% (78/107) the relatedness verdict was based on mutation and copy number data, in 25% (27/107) it was based on mutation data alone and 2% (2/107) on copy number data alone, Fig. 2. Of those that had data on both platforms, 63% (49/78) were concordant. In a further 26% (20/78) one platform was able to assign a verdict of related (5/78) or unrelated (15/78), and the other platform was ambiguous. In 12% (9/78) the results from the two platforms were discordant. Similar numbers of pairs were called ambiguous on both platforms due to either the small numbers of shared breakpoints relative to breakpoints private to one of the tumours or due to shared hotspot mutations in *TP53* or *PIK3CA*, Figs. 2 and 3.

Overall, 65% (20/31) of ipsilateral subsequent cancers in IMPORT HIGH were clonally related to the index tumour and could be considered true recurrences. In contrast, only 32% (7/21) in the lower risk IMPORT LOW cohort were true recurrences (P = 0.003, Fisher’s Exact test). Six/31 (19%) and 1/21 (5%) ipsilateral pairs in IMPORT HIGH and IMPORT LOW, were considered ambiguous, respectively (P = 0.22, Fisher’s Exact test).

As expected, 95% (35/37) of contralateral tumours in IMPORT LOW showed no evidence of a clonal relationship to index tumours and two were classified as ambiguous. However, in IMPORT HIGH only 67% (12/18) of contralateral second tumours were classed as unrelated with 22% (4/18) of contralateral tumours being considered clonally related suggesting they are likely to be metastases rather than new primaries, Figs. 2 and 3.

### Correlation with clinicopathological variables

Of the 52 tumour pairs included in the clonality analysis with subsequent ipsilateral events, 43/52 (83%), 50/52 (96%) and 43/52 (83%) had all data available regarding tumour grade, ER status, and HER2 status respectively. Of the clonally related tumours 17/24 (71%) had the same grade as the index cancer, 21/26 (81%) had the same ER status, and 23/23 (100%) had the same HER2 status, Supplementary Table 1. For clonally unrelated tumours, 7/12 (58%) had the same grade as the index cancer, 14/17 (82%) had the same ER status, and 9/14 (64%) had the same HER2 status.

Using the criteria of Jobsen et al. [5] (the ‘morphology method’, which uses histological criteria only, see methods) to assess relatedness, only 8/27 (30%) ipsilateral cases judged to be related in our genomic analysis would have been classed as such. The majority, 16/27 (59%), would have been classed as unrelated with 3/27 (11%) being impossible to classify. Four/18 (22%) with no genomic evidence of relatedness in our analysis would have been called related using these histological criteria. In total 10/31 (32%) from IMPORT HIGH, and 7/21 (32%) from IMPORT LOW, would have been called related using this method.

### Correlation with outcome

To assess whether development of true recurrences varied with time we dichotomised analysis into subsequent ipsilateral event < 5 years versus > 5 yrs. Of the subsequent ipsilateral tumours diagnosed < 5 years from trial randomisation, 23/35 (66%) were genomically related to the index cancer and 8/35 (23%) were classed as unrelated. In contrast for the tumours diagnosed > 5 years from randomisation, only 4/17 (24%) were related; 10/17 (59%) were unrelated. The association between timing to diagnosis and related or unrelated genomic classification was statistically significant (P = 0.008, Fisher’s exact test). For ipsilateral tumour pairs the median time from randomisation to diagnosis of recurrence was shorter for related tumours: 29 months (range 7–105 months) compared with 66 months (range 6–110 months) for unrelated tumours (P = 0.002, Mann Whitney U test).

The proportion of participants diagnosed with metastatic breast cancer or dying during follow-up by the time of data snapshot is presented in Supplementary Table 2. Similar proportions of those with related, ambiguous and unrelated subsequent tumours experienced these outcomes.

Of the four contralateral cases in which the subsequent tumours were classified as genomically related, three women had either developed distant metastases or died at the time of data snapshot.

### Correlation with spatial mapping

The clinically recorded site of the ipsilateral subsequent event as determined by the treating physician was available from the trial CRF in 48/53 cases. Genomically related tumours were more frequently seen within the index quadrant compared to unrelated cases for both trials (IMPORT HIGH 13/20 (65%), 1/5 (20%); IMPORT LOW, 4/7 (57%) 5/13 (38%) respectively (P = 0.07, Fisher’s Exact Test).

**Fig. 4:**
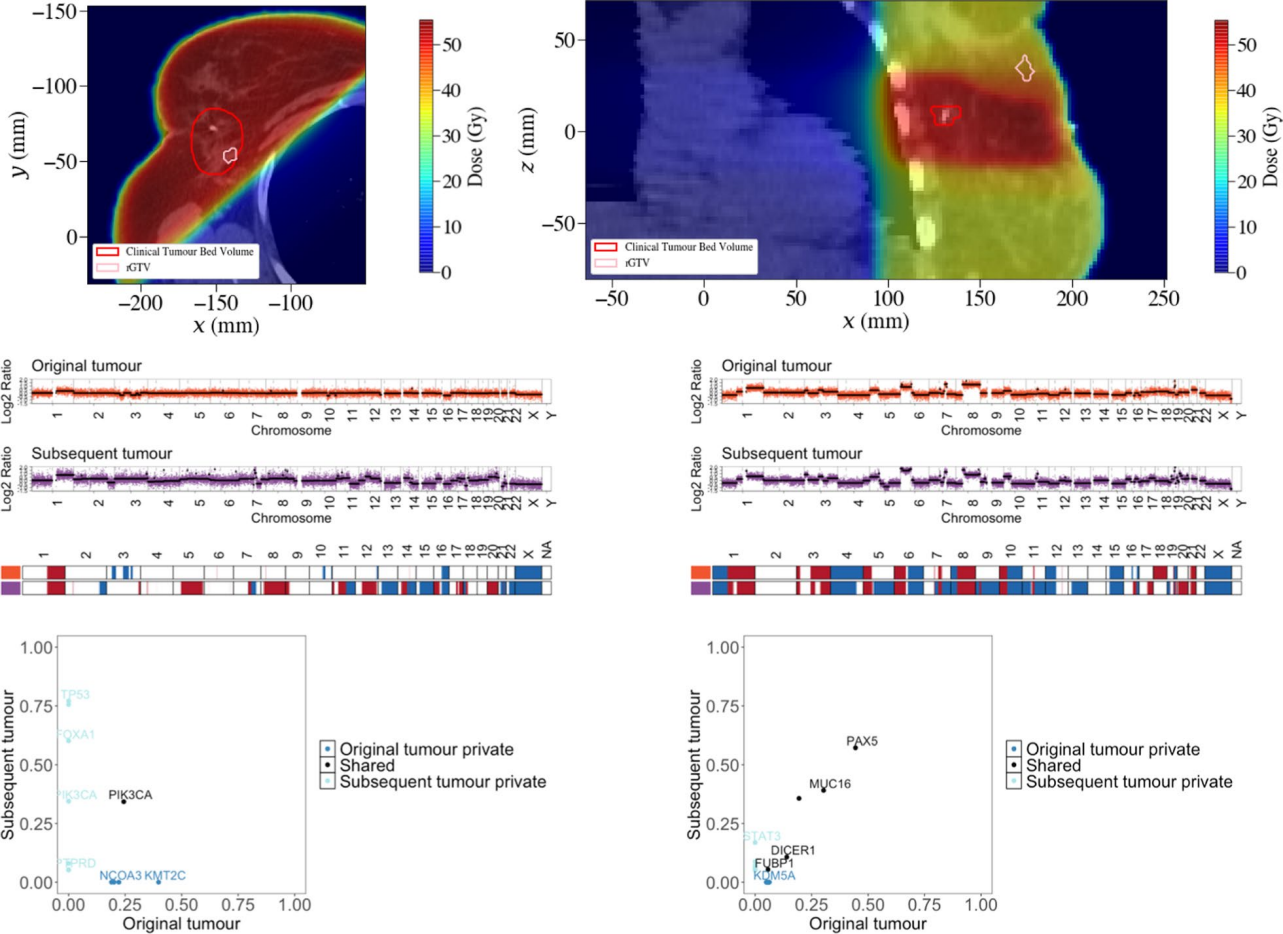
Example **A** cross sectional images from radiotherapy planning CT scans, showing the locations of the original tumour bed, transformed recurrence volume (rGTV), and recurrence sphere, from (1) a case from the IMPORT LOW cohort in which the rGTV was within the original tumour bed, in which the two tumours were genomically unrelated, and (2) a case from the IMPORT HIGH cohort in which the rGTV was located distant to the original tumour bed, in which the two tumours were found to be genomically related. The radiotherapy dose distribution is overlaid as per the colour bars to the right of each image. **B** shows matched original and subsequent tumour copy number profiles, and **C** plots of variant allele fraction of shared and private mutations between the paired tumours

A complete spatial mapping dataset of radiotherapy planning CT, and recurrence CT with the tumour volume outlined, was available for 20 cases from IMPORT HIGH and 6 from IMPORT LOW, Fig. 2. The clinico-computational classification agreed with the Case Report Form spatial classification in 75% of cases (18/24). Although the majority of the genomically related subsequent tumours with full imaging data available were either in the tumour bed (36% -5/14) or the same quadrant as the tumour bed (43% -6/14) there were some tumours that were genomically related but in distant quadrants (21% -3/14). Similarly of those classed as unrelated genomically the majority were found in tumour bed (25% -2/8) or in the same quadrant (50% -4/8) with only two (25% -2/8 (25%) found in a distant quadrant, Fig. 4, Supplementary Figs. 2 and 3. When looking at the absolute centroid distances between the recurrent gross tumour volume and the index tumour bed across both trials, there were no differences between related and unrelated cases, median 40 mm (IQR 18-53 mm) and 37 mm (IQR 22-57 mm), respectively, but the median distance was larger for the ambiguous cases (53 mm, IQR 38-68 mm), Supplementary Fig. 2. Details of relatedness and spatial classifications alongside other metrics from co-registered CT images and trial group for each case are presented in Supplementary Tables 3 and 4.

### Genomic landscape of recurrent breast cancer

Further analysis was undertaken to describe the genomic landscape in this cohort of recurrent breast cancer to identify genomic differences that may predict recurrence. We also compared the genomic changes between the two trial cohorts (lower risk cancers in IMPORT LOW and higher risk in IMPORT HIGH) and finally compared the genomic changes in the index tumours to the paired recurrences.

### Copy number analysis

**Fig. 5:**
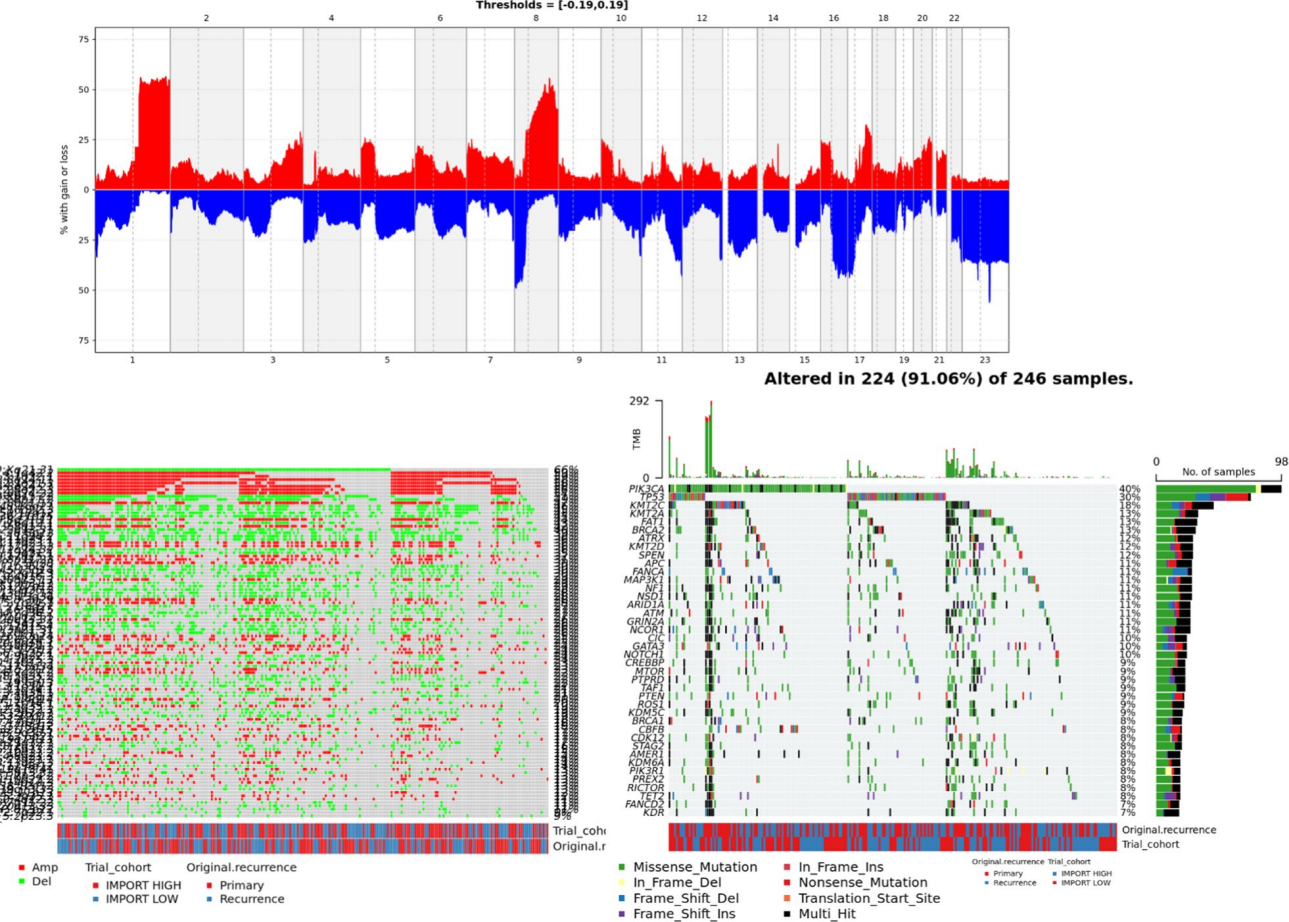
**A** Frequency plot displaying broad regions of copy number gain and loss delineated by GISTIC analysis of the cohort. **B** Oncoplot displaying the top 40 focal copy number abnormalities found in the cohort, by sample. The bars below the plot indicate the status as index or subsequent tumour and trial cohort of each sample. **C** Oncoplot displaying the top 40 mutations found in the cohort, by sample. The bars below the plot indicate the status as index or subsequent tumour and trial cohort of each sample

Copy number data was generated on 52 paired ipsilateral recurrences. There were 105 recurrently occurring regions of focal copy number aberration across both cohorts as defined by GISTIC, Fig. 5. The two most common high-level amplifications in the index tumours were on 8q24.21 (in which the *Myc* proto-oncogene is located) in 41% and 1q21.3 (which harbours the *BCL-9* proto-oncogene) in 33%. These were found at a much higher frequency than TCGA breast cancer samples that were not selected for recurrence, where the frequencies were 15.6% (Fisher’s Exact Test, P = 0.00003) and 5% (Fisher’s Exact Test, P = 6.2 X 10 ^−9^), respectively suggesting these aberrations may be associated with local recurrence.

In order to test the hypothesis that amplifications in index tumours on 8q24.21 and 1q21.3 were associated with local recurrence the frequency of these amplifications was compared in the related and unrelated pairs from IMPORT HIGH. This revealed that 41% of related and only 18% of unrelated pairs had 8q24.21 amplification (P = 0.01, Fisher’s Exact test) supporting our hypothesis. In contrast, there was no difference in the frequency of 1q21.3 amplifications in related and unrelated cases (33% versus 27%, P = 0.28, Fisher’s Exact test).

Considering the two trials, amplification of 10q22.2 was more common in IMPORT HIGH (FDR = 0.025). Otherwise, CNAs were similar across the two trials. There were no recurrent CNAs that were significantly enriched in ipsilateral subsequent tumours compared to the index tumours, and the proportion of the genome that was aberrated, gained, amplified or deleted between original tumours and their paired related subsequent tumours was similar.

### Targeted sequencing analysis

Targeted sequencing data was available from 52 paired ipsilateral recurrences. The median number of mutations per sample was 4 (range 0–292), and top 10 mutated genes across the whole cohort were *PIK3CA* (40%), *TP53* (30%), *KMT2C* (18%), *KMT2A* (13%), *FAT1* (13%), *BRCA2* (13%), *ATRX* (12%), *SPEN* (12%), *KMT2D* (12%) and APC (11%). As expected, *PIK3CA* mutations were more common in IMPORT LOW (29/56 (52%) versus 13/49 (27%)) whilst *TP53* mutations were more common in IMPORT-HIGH (18/49 (37%) versus 13/56 (23%)) although neither of these differences met statistical significance, and *TP53* and *PIK3CA* mutations appeared mutually exclusive (p < 0.01).

There were no genes that were significantly differentially mutated between the original tumours and ipsilateral subsequent tumours, following FDR correction, in the present study.

In order to explore whether the previous radiotherapy had an effect on the genomic changes found in the ipsilateral subsequent recurrences, tumour mutational burden (TMB) was compared between original tumours and their corresponding recurrences, Supplementary Fig. 1. There was a borderline significant decrease in TMB in the recurrent tumours (P = 0.015, McNemar’s test). However, a significantly greater number of large deletions and a higher deletion:insertion ratio were seen in ipsilateral subsequent tumours, all of which developed in likely irradiated breast tissue, irrespective of whether they were true recurrences or new primaries, compared with index cancers (P = 0.021 and P = 0.0074, respectively). There was also a borderline association with higher indel:substitution ratio seen in ipsilateral subsequent tumours (P = 0.051). There were no significant differences seen in mean deletion length between these groups. Additionally, there were no significant differences found between unrelated ipsilateral and contralateral subsequent tumours in deletion:insertion or indel:substitution ratios.

## Discussion

This study analyses the genomic profiles of matched index and subsequent tumour pairs from two large randomized controlled trials—IMPORT-LOW (low-risk breast cancer) and IMPORT-HIGH (higher-risk breast cancer). We found distinct differences in clonal relationships between these cohorts. In IMPORT-LOW, subsequent ipsilateral breast tumours were less likely to be clonally related compared to IMPORT-HIGH, indicating that, in women with low-risk breast cancers, subsequent breast tumours are more likely to be new primaries rather than recurrences. In contrast, most subsequent events in IMPORT-HIGH were true recurrences, despite these patients receiving a higher radiation dose to the tumour bed.

As expected, the time between the original treatment and subsequent diagnosis was longer for unrelated cases than for those considered true recurrences. A higher proportion of tumours diagnosed after five years were genomically unrelated, supporting the notion of new primaries. The intermediate timing of ambiguous cases likely reflects a mix of true recurrences and new primaries. However, the longer follow-up in IMPORT-LOW may confound these findings, as this cohort had fewer true recurrences at the time of analysis. Additionally, the small number of outcome events limited formal analysis of relationships between clonal relatedness and clinical outcomes.

Classification based on CRF showed a higher proportion of subsequent tumours within the index quadrant in the ‘related’ group. Contrary to expectations image registration did not reveal any clear change in spatial pattern to the location of the subsequent tumour relative to the index tumour by clonality verdict. There were similar proportions classed by our clinico-computational method as being in the tumour bed, the index quadrant, and other quadrants, in the genomically related and unrelated groups. Dividing the breast into quadrants is clinically useful but does not necessarily reflect the underlying anatomy of the ductal tree as some ducts may span multiple quadrants. Using the median absolute centroid distance between index and subsequent tumours did also not reveal any difference between related and unrelated pairs. However, the small sample size with complete imaging data and genomic results limits the strength of these conclusions. In IMPORT-LOW, about one-third of participants did not undergo cross-sectional imaging at the time of ipsilateral breast tumour recurrence (IBTR), possibly due to being considered “low risk,” which could introduce bias.

Our findings suggest that some contralateral cancers in high-risk breast cancer patients are likely metastases, a possibility also raised by previous genomic studies [9, 10, 20]. As Begg et al. discuss in their analysis, this conclusion is based on findings of sufficient shared genomic changes that chance occurrence in independent tumours in different individuals is highly unlikely. It could be argued that two independent tumours in one host may have a different probability distribution of shared genomic changes due to host factors, and that the similarities seen here reflect exposure to similar mutational processes rather than clonal origin. The results of a recent mitochondrial DNA analysis support our conclusion however; mitochondrial DNA does not develop hotspot mutations meaning that identical mutations in unrelated tumours are vanishingly unlikely, and their presence in 7/30 bilateral breast cancer pairs in that cohort imply metastasis rather independent development [21]. Epidemiological data also indirectly support this possibility: higher rates of CBC have been reported following breast cancers with features that put them at higher risk of metastasis [22]. The implications of this are important: management could be altered significantly if cases of metastatic spread to the contralateral breast could be reliably detected as part of standard care.

Further genomic analysis revealed that amplification of 8q24.21, which harbours *c-myc*, was more common in tumours with clonal recurrences. This aligns with previous studies showing that *c-myc* amplification is associated with worse outcomes and endocrine resistance in breast cancer [23]. We had speculated that we might see evidence of radiotherapy-related genomic changes, which have been observed in other studies [24, 25] when comparing original tumours to ipsilateral subsequent tumours or original tumours and their paired true recurrences. The higher deletion:insertion and indel:substitution ratios seen here in ipsilateral subsequent tumours are in keeping with previous reporting in radiation-associated second malignancies by Behjati et al. [25], while the stability of small deletion numbers is contrary to the results of Kocakavuk et al. [24]. Contrary to expectations there was a borderline significant reduction in tumour mutational burden between primaries and paired true recurrences. Patterns of change in TMB during cancer evolution have not been studied extensively, and to our knowledge, there has been no prior analysis of this in the setting of cancer local recurrence, but the data that is available suggest that metastases have either stable or higher TMB than their index tumours [26, 27].

Our study supports the finding of others [17, 28] that the classification of ipsilateral subsequent tumours as related or unrelated cannot be determined with high accuracy based on clinico-pathological data alone, thus genomic approaches to assess clonal relatedness are essential [29]. However these methods are not always able to classify tumours as demonstrated by a number of ambiguous pairs in our data. With deeper sequencing we may be able to resolve some of these ambiguous cases using haplotype-specific copy number profiles as described in the recent preprint from Kader et al. [30].

As far as we are aware, this work represents the first published analysis combining genomic relatedness and spatial relationships of ipsilateral subsequent tumours using next-generation sequencing and computational co-registration of imaging. The large sample size (over 2000 participants in each trial) and detailed data on IBTR site which would not be available in a real-world setting, are strengths of this study. An additional strength is the high-quality radiotherapy quality assurance provided by the RTTQA group. However, the small number of local recurrences in both trials limited our ability to draw firm conclusions about outcomes in different subgroups, underscoring the need for validation in larger, independent datasets.

In conclusion our study underscores the significant difference in the likelihood of a subsequent tumour being a true recurrence or a new primary between low- and high-risk breast cancer patients. This distinction has important implications for treatment strategies and long-term monitoring.

## Supplementary Information

Below is the link to the electronic supplementary material.

Supplementary Material 1

Supplementary Material 2

## Data Availability

Data is provided within the supplementary files. Raw sequencing data can be deposited in EGA if accepted.

## References

[1] Darby S, et al. Effect of radiotherapy after breast-conserving surgery on 10-year recurrence and 15-year breast cancer death: meta-analysis of individual patient data for 10,801 women in 17 randomised trials. Lancet. 2011;378:1707–16. 22019144 10.1016/S0140-6736(11)61629-2 PMC3254252

[2] Wapnir IL, et al. Prognosis after ipsilateral breast tumor recurrence and locoregional recurrences in five national surgical adjuvant breast and bowel project node-positive adjuvant breast cancer trials. J Clin Oncol. 2006;24:2028–37. 16648502 10.1200/JCO.2005.04.3273

[3] Bosma SCJ, et al. Very low local recurrence rates after breast-conserving therapy: analysis of 8485 patients treated over a 28-year period. Breast Cancer Res Treat. 2016;156:391–400. 27008183 10.1007/s10549-016-3732-0

[4] Veronesi U, et al. Radiotherapy after breast-conserving surgery in small breast carcinoma: long-term results of a randomized trial. Ann Oncol. 2001;12:997–1003. 11521809 10.1023/a:1011136326943

[5] Jobsen JJ, Struikmans H, Siemerink E, van der Palen J, Heijmans HJ. The clinical relevance of various methods of classifying ipsilateral breast tumour recurrence as either true local recurrence or new primary. Breast Cancer Res Treat. 2022;195:249–62. 35939185 10.1007/s10549-022-06680-7

[6] Vicini FA, et al. The use of molecular assays to establish definitively the clonality of ipsilateral breast tumor recurrences and patterns of in-breast failure in patients with early-stage breast cancer treated with breast-conserving therapy. Cancer. 2007;109:1264–72. 17372920 10.1002/cncr.22529

[7] Masuda S, et al. Analysis of gene alterations of mitochondrial DNA D-loop regions to determine breast cancer clonality. Br J Cancer. 2012;107:2016–23. 23169290 10.1038/bjc.2012.505 PMC3516690

[8] Desmedt C, et al. Uncovering the genomic heterogeneity of multifocal breast cancer. J Pathol. 2015;236:457–66. 25850943 10.1002/path.4540 PMC4691324

[9] Alkner S, et al. Contralateral breast cancer can represent a metastatic spread of the first primary tumor: determination of clonal relationship between contralateral breast cancers using next-generation whole genome sequencing. Breast Cancer Res. 2015;17:102. 26242876 10.1186/s13058-015-0608-x PMC4531539

[10] Klevebring D, et al. Exome sequencing of contralateral breast cancer identifies metastatic disease. Breast Cancer Res Treat. 2015;151:319–24. 25922084 10.1007/s10549-015-3403-6

[11] Coles CE, et al. Partial-breast radiotherapy after breast conservation surgery for patients with early breast cancer (UK IMPORT LOW trial): 5-year results from a multicentre, randomised, controlled, phase 3, non-inferiority trial. Lancet Lond Engl. 2017;390:1048–60. 10.1016/S0140-6736(17)31145-5 PMC5594247 28779963

[12] Coles CE, et al. Dose-escalated simultaneous integrated boost radiotherapy in early breast cancer (IMPORT HIGH): a multicentre, phase 3, non-inferiority, open-label, randomised controlled trial. Lancet. 2023. 10.1016/S0140-6736(23)00619-0. 37302395 10.1016/S0140-6736(23)00619-0

[13] Postoperative radiotherapy for breast cancer: UK consensus statements. 2016. www.rcr.ac.uk 10.1016/j.clon.2017.06.011 28687408

[14] Shaitelman SF, et al. Partial breast irradiation for patients with early-stage invasive breast cancer or ductal carcinoma in situ: an ASTRO clinical practice guideline. Pract Radiat Oncol. 2023;14:112. 37977261 10.1016/j.prro.2023.11.001

[15] Meattini I, et al. European society for radiotherapy and oncology advisory committee in radiation oncology practice consensus recommendations on patient selection and dose and fractionation for external beam radiotherapy in early breast cancer. Lancet Oncol. 2022;23:e21–31. 34973228 10.1016/S1470-2045(21)00539-8

[16] Tsang Y, et al. EP-1784: clinical impact of IMPORT HIGH trial (CRUK/06/003) on breast radiotherapy in the United Kingdom. Radiother Oncol. 2014;111:S282–3. 10.1259/bjr.20150453 PMC4984937 26492402

[17] Lips EH, et al. Genomic analysis defines clonal relationships of ductal carcinoma in situ and recurrent invasive breast cancer. Nat Genet. 2022;54:850–60. 35681052 10.1038/s41588-022-01082-3 PMC9197769

[18] Klein S, Staring M, Murphy K, Viergever MA, Pluim JPW. elastix: a toolbox for intensity-based medical image registration. IEEE Trans Med Imaging. 2010;29:196–205. 19923044 10.1109/TMI.2009.2035616

[19] Harrison, H. P., K. scikit-rt: Toolkit for analysis of radiotherapy data.

[20] Begg CB, et al. Contralateral breast cancers: independent cancers or metastases? Int J Cancer. 2018;142:347–56. 28921573 10.1002/ijc.31051 PMC5749409

[21] Girolimetti G, et al. Mitochondrial DNA analysis efficiently contributes to the identification of metastatic contralateral breast cancers. J Cancer Res Clin Oncol. 2021;147:507–16. 33236215 10.1007/s00432-020-03459-5 PMC7817585

[22] Vichapat V, et al. Tumor stage affects risk and prognosis of contralateral breast cancer: results from a large Swedish-population-based study. J Clin Oncol Off J Am Soc Clin Oncol. 2012;30:3478–85. 10.1200/JCO.2011.39.3645 22927521

[23] Green AR, et al. MYC functions are specific in biological subtypes of breast cancer and confers resistance to endocrine therapy in luminal tumours. Br J Cancer. 2016;114:917–28. 26954716 10.1038/bjc.2016.46 PMC4984797

[24] Kocakavuk E, et al. Radiotherapy is associated with a deletion signature that contributes to poor outcomes in cancer patients. Nat Genet. 2021;53:1088–96. 34045764 10.1038/s41588-021-00874-3 PMC8483261

[25] Behjati S, et al. Mutational signatures of ionizing radiation in second malignancies. Nat Commun. 2016;7:12605. 27615322 10.1038/ncomms12605 PMC5027243

[26] Steindl A, et al. Tumor mutational burden and immune infiltrates in renal cell carcinoma and matched brain metastases. ESMO Open. 2021;6:100057. 33588158 10.1016/j.esmoop.2021.100057 PMC7890370

[27] Gorris MAJ, et al. Paired primary and metastatic lesions of patients with Ipilimumab-treated melanoma: high variation in lymphocyte infiltration and HLA-ABC expression whereas tumor mutational load is similar and correlates with clinical outcome. J Immunother Cancer. 2022;10:e004329. 35550553 10.1136/jitc-2021-004329 PMC9109111

[28] Bollet MA, et al. High-resolution mapping of DNA breakpoints to define true recurrences among ipsilateral breast cancers. J Natl Cancer Inst. 2008;100:48–58. 18159071 10.1093/jnci/djm266

[29] Kader T, Zethoven M, Gorringe KL. Evaluating statistical approaches to define clonal origin of tumours using bulk DNA sequencing: context is everything. Genome Biol. 2022;23:43. 35109903 10.1186/s13059-022-02600-6 PMC8809045

[30] Kader T, et al. Phylogenetic analysis of paired breast carcinomas identifies genetic events associated with clonal recurrence and invasive progression. J Pathol. 2026;268:1–12. 10.1002/path.6461 PMC12699244 41165701

