# Supplementary material for: Genomic and spatial analysis of local recurrences following risk-adapted breast radiotherapy in the IMPORT trials
Source: Breast Cancer Res. 2026 Apr 25;28:108. doi: 10.1186/s13058-026-02232-9 (PMC13267394; doi:10.1186/s13058-026-02232-9)
Supplement: Supplementary file 1 [file 13058_2026_2232_MOESM1_ESM.odt]

Supplementary Methods:

Clinicopathological data:

Clinicopathological data were obtained from the trial database and central pathological review.  Central pathological review was conducted by the trial pathologist (EP) using sections taken from the tumour FFPE blocks; tumour percentage invasive and in situ component, overall cellularity, histological type and grade were obtained from this review. ER status (positive/negative), HER2 status (positive/negative), and presence/absence of an invasive component where only DCIS was found on pathological review were obtained from the trial database and/or histology reports from participating centres. 

DNA extraction:

Formalin-fixed paraffin-embedded (FFPE) blocks from original and subsequent tumours in these cases were obtained from the participating trial centres. Areas of tumour were marked on the blocks by the trial pathologist (EP) and cores were taken from these areas. DNA was extracted from the cores from index and subsequent cancers, using either the Qiagen FFPE DNeasy kit, Qiagen FFPE Allprep kit, or Beckman FormaPure XL kit. Germline DNA was extracted from whole blood samples in those for whom this was available using the Magcore DNA Whole Blood kit.

Cases were excluded from the analysis if blocks from one or both tumours of an index-subsequent tumour pair were not available, if insufficient tumour was present in one or both blocks of the pair, or if insufficient DNA was extracted from one or both cores of the pair for DNA sequencing.

Bioinformatic analysis of targeted sequencing data:

The Genome Research Consortium human build 38 (GRCh38) assembly of the human genome was taken as the reference genome. For each PanCancer FASTQ file, sequencing quality metrics were generated using FastQC17. Alignment was performed using the Burrows-Wheeler Aligner Maximal Exact Matches algorithm (BWA-MEM)18. Potential sample mismatches were screened for using the R package NGSCheckMate19. Variants were called by Mutect220, and annotated using the command line tool Annovar21. Variants called were then filtered to identify true somatic mutations. Variants were removed if they met any of the following criteria: variant allele fraction (VAF) less than 5%; coverage of the locus less than 30; synonymous or intronic variant; present at a frequency of 0.01 or greater in the databases ExAC22, ESP650023 or 1000 Genomes24, or more than 5 times in the gnomAD database. C to T or G to A transitions with VAF less than 10% were also removed, unless present in the COSMIC database25 3 or more times. For samples in which germline sequencing was not available, variants were also removed if listed as germline in the Clinvar database26. 

Hotspot mutations in TP53 and PIK3CA genes that had been removed with these filtering criteria were added back in to the dataset, unless they had been removed from samples without matched germline DNA due to listing as germline in the Clinvar database. 

Bioinformatic analysis of shallow whole genome sequencing data:

Quality metrics and alignments for sWGS data were produced as above. Relative copy number profiles were obtained with QDNAseq (version 1.36.0)27, using a 100 kb fixed bin size. This bin size was used as in previous experience this gave the best balance between sensitivity and noise28. Bin annotations for GRCh38 were created as QDNAseq does not support the GRCh38 assembly. Copy number profiles were filtered out if there were fewer than 1.5 million reads, if the number of segments was greater than 400, or if the observed/expected noise ratio was greater than 50.

Following the application of these quality control criteria there remained a small subset of poor-quality copy number profiles. All copy number plots were therefore assessed visually by an expert (ES). Those that were considered poor quality such that copy number calls may not have been accurate were removed from the cohort.

Downstream genomic analysis:

Further analysis was conducted to understand the genomic landscape of both cohorts more fully. Prior to the mutation analyses, BED files (available in Supplementary Appendix 1) including panels of both IMPORT LOW and IMPORT HIGH were intersected using IRanges R package. The plotmafSummary and oncoplot functions in the Maftools package in R, both using default parameters, were used for visualization of the mutation landscape of all samples[43](https://www.ncbi.nlm.nih.gov/pmc/articles/PMC11137060/#CR43). The somaticInteractions Maftools function, using default parameters, was used for the identification of co-occurring or mutually exclusive genes. 

 

Analysis of CNAs was performed with GISTIC2.029 with the alteration cutoffs set at +/-0.2 for amplifications and deletions, 0.9 for deep amplifications and -1.3 for deep deletions. Alterations were considered if they surpassed a q-value threshold of 0.25 with 99% confidence interval. A threshold of 60% of the chromosome arm was used to distinguish broad from focal events.

Deletion signatures were estimated using the list of somatic mutations identified through targeted sequencing after filtering out germline variants and potential FFPE-induced artifacts, as detailed in the section "Bioinformatic Analysis of Targeted Sequencing Data."  and the copy number aberration (CNA) data obtained as described in the section "Bioinformatic Analysis of Shallow Whole Genome Sequencing Data."

To account for differences in sequencing coverage between CNAs and mutations, the number of aberrations, stratified by variant type, was used as a proxy for burden. For deletions, mutations were further categorized into small deletions (5–15 bp) and large deletions (20 bp to chromosome-arm length or greater) following the methodology outlined by Kocakavuk et al30. Additionally, the deletion / insertion ratio and the indel / substitution ratio were computed as described by Behjati et al31.
The aneuploidy score was determined using the implementation of the Cohen-Sharir et al. method32 within the sigminer R package33 

Comparative analysis of the mutational and copy number landscapes between the following groups was performed: index tumours in the two trial cohorts, index tumours with the TCGA breast cancer cohort, index tumours and their true recurrences, index tumours and ipsilateral subsequent tumours,  indexes with true recurrences and those with unrelated subsequent tumours, and ipsilateral and contralateral unrelated subsequent tumours. The R packages ggplot236 and ggpubr37 were used for visualization of comparative analyses.

Spatial data:

To classify patterns of locoregional failure, a deformable image registration (DIR) workflow was performed using Elastix software38 as part of the scikit-rt Python package39. The DIR workflow performed an initial alignment of the planning CT and recurrence CT using the sternum centroid as identified on both scans, followed by a rigid transformation, and finally, a deformable b-spline transform. The resulting final transform was obtained from the DIR workflow and applied to the recurrence CT sternum and carina structure segmentations to quantify the DIR accuracy. For each patient, the target registration error was calculated as the absolute distance between the centroid of the planning sternum and spatially transformed sternum, and the centroid of the planning carina and spatially transformed carina; these were chosen as anatomical points that were reliably identifiable on CT and relatively unlikely to be affected by anatomical changes over time. The final cohort target registration error for each trial was defined using a global mean of the absolute centroid distance (mm) for the sternum and carina across all patients in the trial with complete spatial data for analysis. Any patient with a target registration error greater than three times the standard deviation of the cohort target registration (, was deemed an outlier and excluded from the analysis due to poor registration performance. The spatial relationship between the rGTV and the CTV TB was described using the absolute centroid distance, calculated as the absolute value of the distance in mm between the centroid of the two structures. 

For cases in both trials, the co-registered imaging datasets were also reviewed by clinicians (CEC and SL) such that the rGTV was viewed on the planning CT along with the outlined tumour bed (CTV TB). In this review, in order to describe the spatial relationship between volumes in a clinically relevant way, the IBTR was given a ‘clinico-computational spatial classification’ as being in either the ‘tumour bed’, ‘quadrant’, or ‘distant’ as per the following criteria. If more than 50% of rGTV area overlapped with that of the outlined CTV TB on any CT slice, or there was any overlap on more than one slice, the IBTR was classified as being in the ‘tumour bed’. If the rGTV was not in the tumour bed, but more than 50% of both the rGTV and CTV TB were in the same quadrant of the breast (upper inner, upper outer, lower inner or lower outer), the IBTR was classified as being in the ‘quadrant’. The IBTR was classified as being ‘distant’ if the two volumes were in different quadrants of the breast, as defined above.

Statistical analysis:

Within the downstream genomic analysis, the two-sided Wilcoxon Rank-Sum test was used for unpaired quantitative data and the Wilcoxon Signed-rank test was used for paired analysis of quantitative data. For independent tests, the two-sided Fisher’s Exact test was used via the fisher.test() function from the R Stats package34. P values were then adjusted for multiple comparisons by the Benjamini and Hochberg false discovery rate (FDR) method35 using the Stats package p.adjust R function. Otherwise, simple descriptive analysis was used as the low numbers precluded more complex analysis. 

Supplemental results

Supplementary Figure 1: Comparison of index tumours and ipsilateral subsequent tumours across both IMPORT trial cohorts: A) Mean deletion length (bp); B) Mean deletion and CNA losses length (bp); C) Number of small deletions; D) Number of large deletions (with CNAs); E) Ratio INDELs/substitutions (with CNAs); F) Ratio deletions/insertions (with CNAs); G) Aneuploidy score; H) Aneuploidy score (deletions)

A)

B)

C)

D)

E)

F)

G)

H)

Supplementary Figure 2: A) Number of cases per each genomic relatedness verdict with clinico-computational spatial categorisations, for cases across both trial cohorts in which both were available; B) Absolute centroid distances (mm) between the original tumour bed CTV and the spatially mapped relapse gross tumour volume (rGTV) vs mean radiation dose to the recurrence volume (Gy), for cases across both trial cohorts in which a genomic relatedness verdict and a complete imaging dataset was available. Tumour bed: subsequent tumour located within the tumour bed of the index cancer; Quadrant: subsequent tumour located within the same quadrant of the breast as the index cancer; Distant: subsequent tumour located in a different quadrant of the breast to the index cancer.

Supplementary Figure 3: A) Matched original and subsequent copy number profiles, and B) plots of variant allele fraction of shared and private mutations between the paired tumours in the two cases classified as genomically unrelated, in which the subsequent tumour was clinico-computationally classified as being in the tumour bed. Variant allele fraction of mutations is as indicated by the colour bars to the right of each heatmap. NB since the number of mutations in case 2) was >150, it is not possible to annotate each individual mutation in the heatmap.

1)

2)

A) 

B) 

A) 

B) 

|  | IMPORT HIGH (n = 49) | IMPORT LOW (n = 58) |
| --- | --- | --- |
| Related | Ambiguous | Unrelated | Total | Related | Ambiguous | Unrelated | Total |
| Original tumour grade | 1 | 1 (50%) | 0 (0%) | 1 (50%) | 2 | 0 (0%) | 1 (8%) | 12 (92%) | 13 |
| 2 | 6 (32%) | 3 (16%) | 10 (53%) | 19 | 4 (14%) | 3 (11%) | 21 (75%) | 28 |
| 3 | 17 (63%) | 5 (19%) | 5 (19%) | 27 | 1 (9%) | 0 (0%) | 10 (91%) | 11 |
| Data missing | 1 (100%) | 0 (0%) | 0 (0%) | 1 | 2 (33%) | 0 (0%) | 4 (67%) | 6 |
| Original tumour ER status | ER positive | 11 (41%) | 4 (15%) | 12 (44%) | 27 | 3 (6%) | 4 (8%) | 42 (86%) | 49 |
| ER negative | 14 (64%) | 4 (18%) | 4 (18%) | 22 | 4 (44%) | 0 (0%) | 5 (56%) | 9 |
| Original tumour HER2 status | HER2 positive | 9 (75%) | 1 (8%) | 2 (17%) | 12 | 0 (0%) | 1 (20%) | 4 (80%) | 5 |
| HER2 negative | 16 (43%) | 7 (19%) | 14/37 (38%) | 37 | 6 (12%) | 3 (6%) | 42 (82%) | 51 |
| Data missing | 0 (0%) | 0 (0%) | 0 (0%) | 0 | 1 (50%) | 0 (0%) | 1 (50%) | 2 |
| Supplementary Table 1: Pathological details of original tumours per trial cohort and subsequent tumour relatedness classification. NB not all data available for all cases. |

|  | Diagnosed with metastatic breast cancer during follow up | Died during follow up |
| --- | --- | --- |
| IMPORT HIGH | IMPORT LOW | IMPORT HIGH | IMPORT LOW |
| Ipsilateral subsequent tumour | Related | 6/20 (30%) | 1/7 (14%) | 7/20* (35%) | 0/7 (0%) |
| Ambiguous | 3/7 (43%) | 0/1 (0%) | 3/7 (43%) | 0/1 (0%) |
| Unrelated | 1/4(25%) | 1/14 (7%) | 1/4 (25%) | 1/14 (7%) |
| Contralateral subsequent tumour | Related | 2/4 (50%) | N/a | 1/4 (25%) | N/a |
| Ambiguous | 0/2 (0%) | 0/2 (0%) | 0/2 (0%) | 0/2 (0%) |
| Unrelated | 0/12 (0%) | 3/33 (9%) | 0/12 (0%) | 5/33† (15%) |
| Supplementary Table 2: Clinical outcomes for participants per trial cohort and subsequent tumour relatedness classification. NB: the median follow-up for IMPORT HIGH trial 74 months, and the median follow-up for IMPORT LOW trial 120 months. *2 without a diagnosis of metastatic breast cancer but with uncontrolled local disease; †2 without a diagnosis of metastatic breast cancer and with a non-breast cancer cause of death. |

| Trial Group | Genomic relatedness verdict | Case Report Form classification | Clinico-computational classification | Absolute centroid distance (mm) | Mean dose to recurrence volume (Gy) | Tumour bed CTV (TB_CTV) volume (mm3) | Recurrence (r_GTV) volume (mm3) |
| --- | --- | --- | --- | --- | --- | --- | --- |
| Control | Ambiguous | Index quadrant | Tumour bed | 29.5 | 51.5 | 19811 | 8096 |
| Test Group 1 | Related | Index quadrant | Tumour bed | 4.5 | 48.6 | 7425 | 2160 |
| Control | Related | Distant breast | Index quadrant | 35.3 | 41.2 | 12462 | 3056 |
| Control | Unrelated | Index quadrant | Index quadrant | 51.9 | 43.5 | 3780 | 568 |
| Test Group 2 | Ambiguous | Distant breast | Distant breast | 65.5 | 35.9 | 21601 | 3976 |
| Test Group 2 | Ambiguous | Index quadrant | Index quadrant | 40.6 | 41.2 | 7132 | 5456 |
| Control | Related | Index quadrant | Tumour bed | 10.0 | 55.4 | 58009 | 7936 |
| Test Group 1 | Unrelated | N/a | Index quadrant | 37.4 | 42.6 | 38861 | 2984 |
| Control | Unrelated | Distant breast | Distant breast | 77.1 | 39.6 | 4182 | 680 |
| Test Group 2 | Related | N/a | Index quadrant | 18.6 | 17.7 | 3535 | 48 |
| Test Group 2 | Unrelated | Distant breast | Distant breast | 72.4 | 38.0 | 5768 | 3760 |
| Test Group 2 | Related | Index quadrant | Distant breast | 51.6 | 40.6 | 31690 | 376 |
| Test Group 2 | Related | Distant breast | Distant breast | 53.6 | 35.8 | 29512 | 192 |
| Test Group 2 | Related | Index quadrant | Tumour bed | 12.4 | 52.1 | 34169 | 2632 |
| Test Group 1 | Related | N/a | Index quadrant | 72.4 | 34.1 | 82721 | 175808 |
| Test Group 2 | Related | Index quadrant | Tumour bed | 18.1 | 51.5 | 105099 | 53880 |
| Test Group 1 | Related | Index quadrant | Tumour bed | 38.2 | 46.8 | 77096 | 45088 |
| Test Group 2 | Related | Distant breast | Index quadrant | 41.6 | 38.1 | 3243 | 1832 |
| Test Group 2 | Related | Index quadrant | Distant breast | 61.2 | 38.0 | 27223 | 12984 |
| Control | Related | Index quadrant | Index quadrant | 46.7 | 40.8 | 14225 | 4088 |
| Supplementary Table 3: Trial group, genomic relatedness verdict, spatial classifications, and relevant metrics of the relationship between subsequent tumours and the corresponding index tumour and radiotherapy dose distribution, in cases from the IMPORT HIGH cohort with both a genomic relatedness verdict and a full spatial mapping dataset. NB since the clinico-computational classification distinguishes between the index tumour bed and wider index quadrant whilst the Case Report Form spatial classification does not, when comparing the two spatial classifications these two categories in the former were considered together to be analogous to the index quadrant category in the latter. |

| Trial Group | Genomic relatedness verdict | Case Report Form classification | Clinico-computational classification | Absolute centroid distance (mm) | Mean dose to recurrence volume (Gy) | Tumour bed CTV (TB_CTV) volume | Recurrence (r_GTV) volume |
| --- | --- | --- | --- | --- | --- | --- | --- |
| Control | Unrelated | Distant breast | Index quadrant | 35.6 | 35.9 | 27985 | 1576 |
| Control | Unrelated | Index quadrant | Tumour bed | 16.9 | 40.2 | 39542 | 416 |
| Test Group 1 | Related | Index quadrant | Index quadrant | 61.2 | 32.9 | 25561 | 440 |
| Test Group 2 | Unrelated | Distant breast | Tumour bed | 18.7 | 40.1 | 14360 | 568 |
| Control | Unrelated | Index quadrant | Index quadrant | 23.7 | 39.9 | 11379 | 64 |
| Test Group 1 | Ambiguous | Distant breast | Distant breast | 74.8 | 41.0 | 32206 | 488 |
| Supplementary Table 4: Trial group, genomic relatedness verdict, spatial classifications, and relevant metrics of the relationship between subsequent tumours and the corresponding index tumour and radiotherapy dose distribution, in cases from the IMPORT LOW cohort with both a genomic relatedness verdict and a full spatial mapping dataset. |
